# Pertussis serology: assessment of IgG anti-PT ELISA for replacement of the CHO cell assay*

**DOI:** 10.1111/j.1600-0463.2010.02664.x

**Published:** 2010-12

**Authors:** TINE DALBY, CHARLOTTE SØRENSEN, JESPER WESTPHAL PETERSEN, KAREN ANGELIKI KROGFELT

**Affiliations:** 1 Department of Microbiological Surveillance and Research, Statens Serum InstitutCopenhagen, Denmark; 2 Quality Control Department, Statens Serum InstitutCopenhagen, Denmark; 3 Bacterial Vaccine Department, Statens Serum InstitutCopenhagen, Denmark

**Keywords:** *Bordetella pertussis*, ELISA, antibody, human

## Abstract

Dalby T, Sørensen C, Petersen JW, Krogfelt KA. Pertussis serology: assessment of IgG anti-PT ELISA for replacement of the CHO cell assay. APMIS 2010; 118: 968–72.

Two types of serological assays are commonly used for the assessment of pertussis vaccine-induced antibodies; the Chinese hamster ovary cell (CHO cell) assay and the immunoglobulin G (IgG) anti pertussis toxin (PT) enzyme-linked immunosorbent assay (IgG anti-PT ELISA). Recently, both the techniques have been modified to improve performance with sera with interfering activity (CHO cell assay) or with heat-treated sera (IgG anti-PT ELISA). These two improved techniques were compared by the analysis of 100 individual serum samples from a previous clinical trial and 213 sera from a longitudinal serum collection from 20 Danish adults recently vaccinated with the Danish acellular pertussis vaccine. The comparison showed a significant linear correlation between the results of the two assays with a p-value of <0.0001 for the 100 individual samples. We, therefore, conclude that the improved IgG anti-PT ELISA can be used as a replacement for the often troublesome and time-consuming CHO cell assay for the measurement of vaccine-induced human antibodies to PT.

In clinical trials of pertussis vaccines, the induced pertussis antibodies are often measured using both an indirect immunoglobulin G (IgG) anti pertussis toxin (PT) enzyme-linked immunosorbent assay (IgG anti-PT ELISA) and a Chinese hamster ovary cell (CHO cell) assay (1–3). Both the methods quantify the binding of human antibodies to PT, but the molecular abilities of the measured antibodies are different, and the antibody-binding sites could possibly be different as well. Thus, in the IgG anti-PT ELISA, the PT is immobilized on a plastic surface and the amount of human IgG antibodies binding to the toxin is measured colorimetrically (4). In the CHO cell assay, neutralization of the biological activity of the PT by human antibodies is evaluated by the inhibition of toxin-mediated clustering of CHO cells (5). In the IgG anti-PT ELISA, the anti-PT antibodies are quantified by automated reading of the amount of produced colour, whereas in the CHO cell assay, the neutralizing anti-PT antibodies are quantified by the use of a dilution series and evaluation of the clustering of the CHO cells by microscopy.

IgG anti-PT ELISA is also frequently used for diagnostic and seroepidemiological purposes (6, 7) and the CHO cell assay is also used for potency testing of pertussis vaccines (8) and for the analysis of residual toxicity in pertussis toxoid vaccines (9).

In a routine diagnostic setting, IgG anti-PT ELISA is easy to perform and a large number of samples can be analysed simultaneously and automatically. Moreover, the availability of an international standard serum (10) has made standardizations of the assay possible. The CHO cell assay, however, is time consuming compared with the IgG anti-PT ELISA, and being an assay involving living cells, fairly large day-to-day variations may occur. The results from the CHO cell assay are dependent on the toxicity of the PT used in the assay (11), and so far, a common standard for PT preparations is not available (12).

Previous studies have shown a good correlation between the IgG anti-PT ELISA and the CHO cell assay (2, 10, 13–18). However, both the IgG anti-PT ELISA and the CHO cell assay have been altered recently. The CHO cell assay was improved by precoating of microplates with foetal calf serum to avoid problems occurring from sera with interfering activity (5). The IgG anti-PT ELISA was improved by addition of a blocking step including powdered skimmed milk to avoid problems occurring when heat-treated sera were tested (4).

In this study, we compared these two recently altered analyses to establish whether the improved IgG anti-PT ELISA can replace the CHO cell assay for quantification of vaccine-induced human antibodies to PT.

## Materials and methods

### Serum samples

A total of 100 serum samples from a previous clinical trial of pertussis combination vaccines (19) were selected according to previously measured CHO cell assay results to represent a broad range of anti-PT titres. In addition, 213 sera from 20 Danish adults who were recently vaccinated with a combination vaccine including acellular PT (DiTeKiPol Booster, Statens Serum Institut, Copenhagen, Denmark) were analysed; 4–13 samples were available from each of those 20 individuals (20).

### CHO cell assay

The CHO cell assay was performed as described by Østergård et al. (5). In brief, 50 μL of a dilution series of serum (1:2 to 1:4096) was mixed with 0.25 ng of PT (Statens Serum Institut, Copenhagen, Denmark) in a microplate and subsequently, 100 μL of CHO cells in culture medium (1 × 10^5^ cells/mL) was added. After incubation, the plates were dried and dyed and clustering of the cells was evaluated microscopically. To avoid interfering activity from frozen samples, the microplates were precoated with 40% foetal calf serum. Results are expressed in titres as the reciprocal of the highest serum dilution resulting in 100% neutralization of the clustering effect of the toxin.

### Enzyme-linked immunosorbent assay

The IgG anti-PT ELISA assay is an in-house developed assay described previously (4). In brief, the assay is an indirect ELISA using 0.2 μg/mL of PT as coating antigen. Sera were analysed in a 1000-fold dilution, and IgG results were expressed as IU/mL according to the WHO International Standard Pertussis Antiserum (National Institute for Biological Standards and Control, Potters Bar, UK, code: 06/140). To avoid false-positive results from heat-treated sera, 1% powdered skimmed milk was included in the blocking buffer and 0.1% powdered skimmed milk was included in the sample dilution buffer.

### Statistical analysis

Results from the CHO cell assay were compared with results from the IgG anti-PT ELISA assay using *Pearson’s product moment* correlation coefficient on log_10_-transformed values. This statistical analysis was calculated only for the 100 individual samples, as the 213 additional samples from the 20 vaccinated persons were not independent.

## Results

**Fig. 1:**
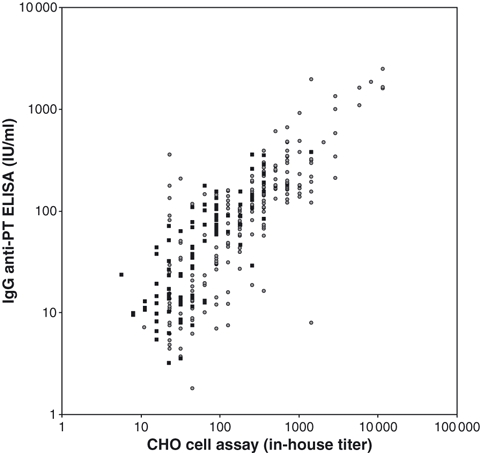
Correlation between immunoglobulin G anti pertussis toxin enzyme-linked immunosorbent assay and Chinese hamster ovary cell assay. Black squares indicate samples from 100 individuals. Grey circles indicate 213 samples from 20 individuals.

When comparing the two analyses, the results are clearly correlated (Fig. 1). The data from the 313 samples tested showed a very good correlation between the two methods, and only a few outliers were observed.

A statistical analysis of the 100 independent samples gave a correlation factor of 0.80 with a p-value of <0.0001.

## Discussion

Human antibodies against PT are conventionally measured by two very different methods: the CHO cell assay and the IgG anti-PT ELISA. The CHO cell assay is based on the detection of toxin-neutralizing antibodies, whereas the ELISA measures the direct binding of antibody to the toxin. However, antibody titres obtained by these two assays display a linear correlation. This correlation has previously been shown for pertussis toxin antibodies induced by acellular pertussis vaccination (2, 13, 14, 17, 21), by whole-cell pertussis vaccination (14), by *Bordetella pertussis* infections (16) and in general (10, 18).

Both methods have been modified during the years; nevertheless, our study shows that the correlation was seemingly unaffected. Diverging results were observed for a few sera, and both combinations of a high result in one assay and a low result in the other assay were seen. Such aberrant results have also been observed previously (14), and the reason for this remains unknown. The general practical difficulties of the CHO cell assay could, however, be a likely explanation.

The CHO cell assay and the IgG anti-PT ELISA were seen to produce correlating results. Although the mechanisms behind the two methods are very different, both involve the binding of specific antibodies to PT. In the case of IgG anti-PT ELISA, only IgG antibodies binding directly to the adsorbed PT are measured, whereas the binding of IgA or IgM is not. In the CHO cell assay, the antibodies should not only bind to the toxin, but also neutralize the effect of the toxin in clustering of the CHO cells. Thus, the avidity and function of the antibodies play an important role in the CHO cell assay, but the assay does neither measure the amount of antibodies nor assess the class of antibodies involved in the neutralization. The observed correlation between the two methods could imply that IgG is either the major factor contributing to neutralization, or that the induced PT antibodies are predominantly of the IgG class. The latter hypothesis is underlined by results from studies of both whole-cell and acellular pertussis vaccines showing either a missing or a modest post-vaccination increase in IgA anti-PT antibodies compared with the increase in IgG anti-PT antibodies (21–25). Moreover, the IgM anti-PT response was found to be negligible both after acellular pertussis vaccination (22) and after whole-cell pertussis vaccination (25).

The correlation between the CHO cell assay and the IgG anti-PT ELISA has also been shown using sera from individuals with confirmed *B. pertussis* infection (16), where the immune response include not only IgG, but also IgA and IgM (26, 27). However, after natural infection, the IgG anti-PT infection response has been shown to be stronger in comparison with the IgA and IgM responses (28). Thus, it would seem that the PT neutralization effect at the CHO cell assay is mainly because of IgG anti-PT antibodies – either because of a specific function of the IgG anti-PT antibodies, or because of the major presence of IgG anti-PT in comparison with IgM and IgA anti-PT antibodies both after pertussis vaccination and after pertussis infection. The CHO cell assay using human serum has been tested previously for use as a diagnostic tool for pertussis, but it was concluded that an ELISA was more sensitive (16). A different approach using the CHO cell assay for pertussis diagnosis based on nasopharyngeal secretions has also been reported (29). In this study, only post-vaccination sera were tested as the CHO cell assay with human serum is now only used for assessment of post-vaccination antibody responses.

## Conclusion

In this study, we show that the measurement of human post-vaccination antibodies against PT by use of two very different methods provides similar results: the IgG anti-PT ELISA measuring the antibody quantity as IU/mL and the CHO cell assay measuring the antibody quantity as arbitrary antibody titres. When comparing the practical aspects of the two methods, the IgG anti-PT ELISA is by far the easiest, fastest and most reproducible method. Moreover, results from IgG anti-PT ELISA can be standardized using the WHO International Standard Serum (10), whereas there is currently no standardization consensus of the CHO cell assay (11, 12). Therefore, when assessing vaccine-induced human antibodies to PT, IgG anti-PT ELISA should be preferred over the CHO cell assay.
